# Patients With Diabetes at High Bleeding Risk With 1-Month Dual Antiplatelet Therapy: Onyx ONE Clear Results

**DOI:** 10.1016/j.jscai.2022.100441

**Published:** 2022-08-25

**Authors:** Elvin Kedhi, Stephan Windecker, Azeem Latib, Ajay J. Kirtane, David Kandzari, Roxana Mehran, Matthew J. Price, Alexandre Abizaid, Daniel I. Simon, Azfar Zaman, Franco Fabbiocchini, Charlies Tie, Arnoud van ‘t Hof, Jose M. de la Torre Hernandez, Christopher J. Hammett, Brent McLaurin, Srinivasa Potluri, Timothy Smith, Ron Waksman, Michael Ragosta, Maria Parke, Te-Hsin Lung, Gregg W. Stone

**Affiliations:** a University of Brussels, Brussels, Belgium; b Silesian Medical University, Katowice, Poland; c University Hospital, University of Bern, Bern, Switzerland; d Montefiore Medical Center, New York, New York; e Columbia University Irving Medical Center/NewYork-Presbyterian Hospital, New York, New York; f Cardiovascular Research Foundation, New York, New York; g Piedmont Heart Institute, Atlanta, Georgia; h The Zena and Michael A. Weiner Cardiovascular Institute, Icahn School of Medicine at Mount Sinai, New York; i Scripps Clinic, La Jolla, California; j Instituto do Coracao, São Paulo, Brazil; k University Hospitals Cleveland Medical Center, Cleveland, Ohio; l Freeman Hospital and Newcastle University, Newcastle upon Tyne, United Kingdom; m Centro Cardiologico Monzino IRCCS, Milano, Italy; n St Andrew's Hospital, Adelaide, Australia; o Maastricht University Medical Center, Maastricht, the Netherlands; p Marques de Valdecilla University Hospital, IDIVAL Santander, Spain; q Royal Brisbane and Women’s Hospital, Brisbane, Australia; r AnMed Health Medical Center, Anderson, South Carolina; s The Heart Hospital Baylor Plano, Plano, Texas; t The Christ Hospital, Cincinnati, Ohio; u MedStar Washington Hospital Center, Washington, DC; v University of Virginia Medical Center, Charlottesville, Virginia; w Medtronic, Santa Rosa, California

**Keywords:** diabetes mellitus, high bleeding risk, percutaneous coronary intervention, zotarolimus-eluting stent

## Abstract

**Background:**

Patients with diabetes mellitus (DM) are at a higher risk of ischemic events compared with patients without DM. Percutaneous coronary intervention (PCI) with the Resolute Onyx zotarolimus-eluting stent (ZES) followed by 1-month dual antiplatelet therapy (DAPT) is safe and effective in patients with high bleeding risk. However, outcomes in patients with DM are not fully understood.

**Methods:**

Onyx ONE Clear was a prospective, multicenter study that included patients receiving the Resolute Onyx ZES during PCI and 1-month DAPT. The primary end point was a composite of cardiac death (CD) or myocardial infarction from 1 month to 12 months.

**Results:**

Among the Onyx ONE Clear population (N = 1506), 39% had DM. Patients with DM had a higher incidence of hypertension, hyperlipidemia, and previous PCI and a higher body mass index than patients without DM. Patients with diabetes were also younger, more likely to be anemic, and experience renal failure. After adjusting for baseline differences between the groups, the Kaplan–Meier rates of CD or myocardial infarction (9.3% vs 6.1%; *P* = .122, unadjusted *P* = .010) and target lesion failure (10.2% vs 7.7%; *P* = .294, unadjusted *P* = .056) between 1 month and 12 months were not significantly different in patients with and without DM. The rates of target lesion revascularization were also similar in both groups, and stent thrombosis was very low and comparable in both arms after adjusting for baseline differences. Non-CD and bleeding were more frequent in patients with DM.

**Conclusions:**

Patients with diabetes treated with the Resolute Onyx ZES followed by 1-month DAPT had favorable 12-month ischemic outcomes after accounting for baseline differences between patients with and without DM, supporting the safety and efficacy of the treatment in selected patients with DM at high bleeding risk.

## Highlights

• Diabetic patients are at high risk of ischemic events after coronary intervention.
• Shortened dual antiplatelet therapy after intervention is an emerging strategy.
• Diabetic patients received the Onyx stent and short dual antiplatelet therapy.
• Diabetic patients had acceptable 1-year outcomes.

## Central Illustration

**Figure:**
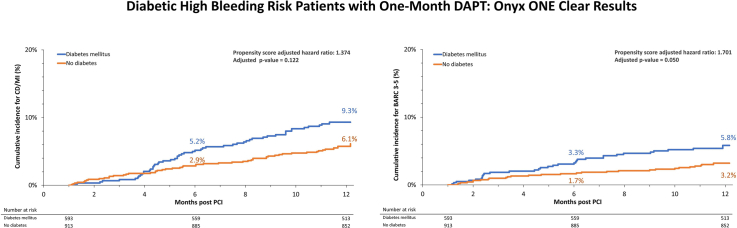

## Introduction

The 1-month duration of dual antiplatelet therapy (DAPT) after percutaneous coronary intervention (PCI) with a drug-eluting stent (DES) or drug-coated stent in patients at high bleeding risk (HBR) has been investigated in several randomized trials and nonrandomized studies.^1^, ^2^, ^3^, ^4^ The Onyx ONE DAPT program demonstrated that Resolute Onyx zotarolimus-eluting stents (ZESs) are safe and effective in a complex patient population with HBR, who received 1-month DAPT after PCI.^1^^,^^4^ However, the optimal duration of DAPT after PCI, particularly for patients with additional comorbidities, is unknown. Patients with diabetes mellitus (DM) and complex lesion anatomy are at a high risk of ischemic events compared with patients without DM.^5^ Furthermore, data on clinical outcomes after current-generation DES implantation in patients with DM and HBR are limited. In this report, we present results from a prespecified subgroup analysis of patients at HBR with versus without DM in the Onyx ONE Clear study who were treated with 1-month DAPT after implantation with the Resolute Onyx ZES.

## Methods

### Study design

Onyx ONE Clear was a prospective, multicenter, single-arm international study that included patients at HBR who received the Resolute Onyx ZES either from the Onyx ONE randomized controlled trial or Onyx ONE US/Japan cohort. Eligible patients were required to adhere to 1-month DAPT and be “clear” of adverse events 1 month after PCI that would necessitate discontinuation of DAPT.^1^ Patients were enrolled with planned PCI if they met at least 1 of the HBR criteria listed in Supplemental Table S1. This analysis compared clinical outcomes in patients with and without DM from the Onyx ONE Clear study. The Onyx ONE Clear study was conducted in accordance with the tenets of the Declaration of Helsinki and was approved by the institutional review board or ethics committee at each enrollment center.

### Study procedures

Patients underwent PCI with implantation of the Resolute Onyx ZES as described previously.^1^ Patients were required to adhere to 1 month of DAPT (75-100 mg aspirin and the standard daily dose of P2Y_12_ inhibitor) after PCI, followed by single antiplatelet therapy (SAPT) with or without an oral anticoagulant (OAC) for the duration of the study. Patients on OAC from the time of the procedure were permitted to receive SAPT and OAC after PCI and continue that treatment for the duration of the study. The Onyx ONE Clear study included only patients who were free of the following events in the first month after PCI that would prevent the cessation of DAPT: myocardial infarction (MI, excluding periprocedural MI), repeat PCI or coronary artery bypass graft (CABG), stroke, definite/probable stent thrombosis (ST), or death, hence 1 month “clear.” Patients receiving an alternative nonstudy stent were excluded from the primary analysis. Clinical outcomes were assessed from 1 month to 12 months after the procedure.

### Study end points

The primary end point of the Onyx ONE Clear study was the composite rate of cardiac death (CD) or MI from 1 month to 12 months.^1^ Patients were required to be on DAPT for the entirety of the first month after PCI, defined as no interruption of aspirin of the P2Y_12_ inhibitor or aspirin for >3 cumulative days during the first month. The secondary end points were rates of all-cause death, CD, MI, clinically driven target lesion revascularization, target lesion failure (the composite of CD, target vessel MI, or target lesion revascularization), definite/probable ST, stroke, and bleeding. MI was defined according to the Third Universal Definition of Myocardial Infarction^6^ and ST according to the Academic Research Consortium.^7^ Bleeding events were defined according to the Bleeding Academic Research Consortium (BARC).^8^ Device, lesion, and procedural success were also examined. Lesion success was defined as the attainment of <30% residual stenosis and Thrombolysis In Myocardial Infarction 3 flow. Moreover, device success was based on lesion success with the assigned study device, and procedural success was based on lesion success and the absence of in-hospital major adverse cardiac events.

### Statistical analysis

Categorical data, reported as percentages (counts), were compared between patients with and without DM using the Fisher exact test. Continuous data, reported as mean ± SD, were compared between the groups using the 2-samples *t-*test. The Kaplan–Meier (KM) estimator method was used to estimate event rates (%) over time. The comparison of clinical outcome rates were calculated from a Cox regression in which an outcome was regressed on the status of diabetes and propensity scores based on age, body mass index (BMI), previous PCI, previous CABG, hyperlipidemia, hypertension, atrial fibrillation, serum creatinine levels, multivessel coronary artery disease, Canadian Cardiovascular Society angina class, minimum baseline reference vessel diameter, and maximum lesion length as the confounding variables. The C-statistic was equal to 0.6272. The outcome variable CD or MI was regressed on the binary variable DM and the estimated propensity score.

A multivariable Cox regression analysis was performed to assess independent correlates of CD/MI and target lesion failure using a stepwise selection of significant risk factors. Variables included age (continuous), sex, hypertension, DM, BMI, hyperlipidemia, previous MI, previous PCI, previous CABG, previous stroke, a history of atrial fibrillation, acute coronary syndrome, Canadian Cardiovascular Society grading of angina pectoris II-IV, anemia or transfusion, renal insufficiency, multivessel disease, smallest reference vessel diameter, total lesion length, femoral vascular access, and complex PCI.^9^ A *P* value of <.1 was required to enter and a *P* value of >.1 was required to be removed, except DM, which was forced into the model. Statistical analyses were performed using SAS version 9.4 (SAS Institute).

## Results

### Patients and procedures

Among the 1506 patients who were event free 1 month after PCI, 593 (39.4%) had DM with 206 (13.7%) treated with insulin, and 913 (60.6%) did not have DM. Compared with those patients without DM, patients with DM had a higher BMI, were more likely to present with hypertension, hyperlipidemia, previous PCI, previous CABG, and multivessel disease, and were more likely to have a creatinine clearance of <40 mL/min. However, patients with DM were younger and less likely to experience atrial fibrillation (Table 1).

**Table 1:** Baseline characteristics.

	DM (n = 593)	No DM (n = 913)	*P*
Age, y	72.9 ± 9.8	74.7 ± 9.3	<.001
Female sex	32.9 (195)	32.0 (292)	.735
Body mass index, kg/m^2^	29.5 ± 6.1	27.4 ± 5.3	<.001
Hypertension	89.5 (531)	80.4 (734)	<.001
Hyperlipidemia	77.2 (458)	69.3 (633)	<.001
Previous MI	28.7 (170)	24.8 (226)	.094
Previous PCI	36.1 (214)	26.4 (241)	<.001
Previous CABG	16.9 (100)	10.3 (94)	<.001
Atrial fibrillation	31.5 (187)	38.2 (349)	.008
Stroke/TIA	14.7 (87)	13.7 (125)	.596
Peripheral vascular disease	12.3 (73)	9.5 (87)	.088
Multivessel disease (≥2)	57.5 (341)	44.9 (410)	<.001
Left ventricular ejection fraction ≤35%	13.6% (59)	12.3% (85)	.583
Renal failure (creatinine clearance <40 mL/min)	18.4% (109)	8.7% (79)	<.001
Clinical presentation			
Silent ischemia	11.8 (67)	10.2 (89)	.386
Chronic coronary syndrome	38.3 (218)	42.0 (366)	.170
Acute coronary syndrome	49.9 (284)	47.8 (417)	.451
Unstable angina	23.2 (132)	22.5 (196)	.748
NSTEMI	22.8 (130)	21.0 (183)	.433
STEMI	3.9 (22)	4.4 (38)	.688

Values are presented as % (n) or mean ± SD.

CABG, coronary artery bypass grafting; DM, diabetes mellitus; MI, myocardial infarction, NSTEMI, non–ST-segment elevation myocardial infarction; PCI, percutaneous coronary intervention; TIA, transient ischemic attack; STEMI, ST-segment elevation myocardial infarction.

Procedural characteristics are provided in Table 2. All patients received the Resolute Onyx ZES only. Patients with DM were more likely to have a smaller reference vessel diameter than patients without DM. Vascular access was more commonly achieved through the femoral artery in patients with DM than in patients without DM. Although the mean number of treated vessels and lesions was similar between the groups, the number of stents per patient and the total stent length were greater in patients with DM.

**Table 2:** Procedural characteristics.

	DM (n = 593 patients) (n = 786 lesions)	No DM (n = 913 patients) (n = 1184 lesions)	*P*
Vascular access			<.001
Radial	59.9 (370)	69.7 (653)	
Femoral	40.0 (247)	29.3 (275)	
Lesion location			
Left main	0.8 (5)	1.5 (14)	.345
Left anterior descending	50.6 (300)	53.7 (490)	.246
Left circumflex	30.7 (182)	26.0 (237)	.052
Right coronary artery	33.1 (196)	34.9 (319)	.470
Bypass graft	5.9 (35)	3.0 (27)	.007
B2/C Lesion class	79.8 (627)	77.8 (913)	.312
Bifurcation (site reported)	10.6 (92)	12.1 (157)	.373
In-stent restenosis	4.0 (35)	2.5 (33)	.060
Chronic total occlusion (site reported)	2.5 (22)	2.4 (31)	.888
Lesion length ≥20 mm (diffuse)	43.0 (324)	39.6 (450)	.152
Moderate or severe calcification	52.3 (407)	48.4 (562)	.095
Preprocedural RVD, mm	2.78 ± 0.47	2.84 ± 0.49	.008
Preprocedural diameter stenosis, %	68.90 ± 13.33	67.91 ± 13.23	.106
Lesion length, mm	21.49 ± 13.95	20.30 ± 12.35	.057
No. of vessels treated	1.21 ± 0.44	1.19 ± 0.43	.363
No. of treated lesions	1.33 ± 0.60	1.29 ± 0.59	.197
Mean No. of stents			
Per patient	1.76 ± 1.09	1.63 ± 0.95	.025
Per lesion	1.20 ± 0.51	1.15 ± 0.41	.023
Total stent length, mm			
Per patient	39.1 ± 28.7	35.5 ± 24.6	.013
Per lesion	26.7 ± 15.9	25.1 ± 12.7	.014

Values are presented as % (n) or mean ± SD.

DM, diabetes mellitus; RVD, reference vessel diameter.

**Figure 1:**
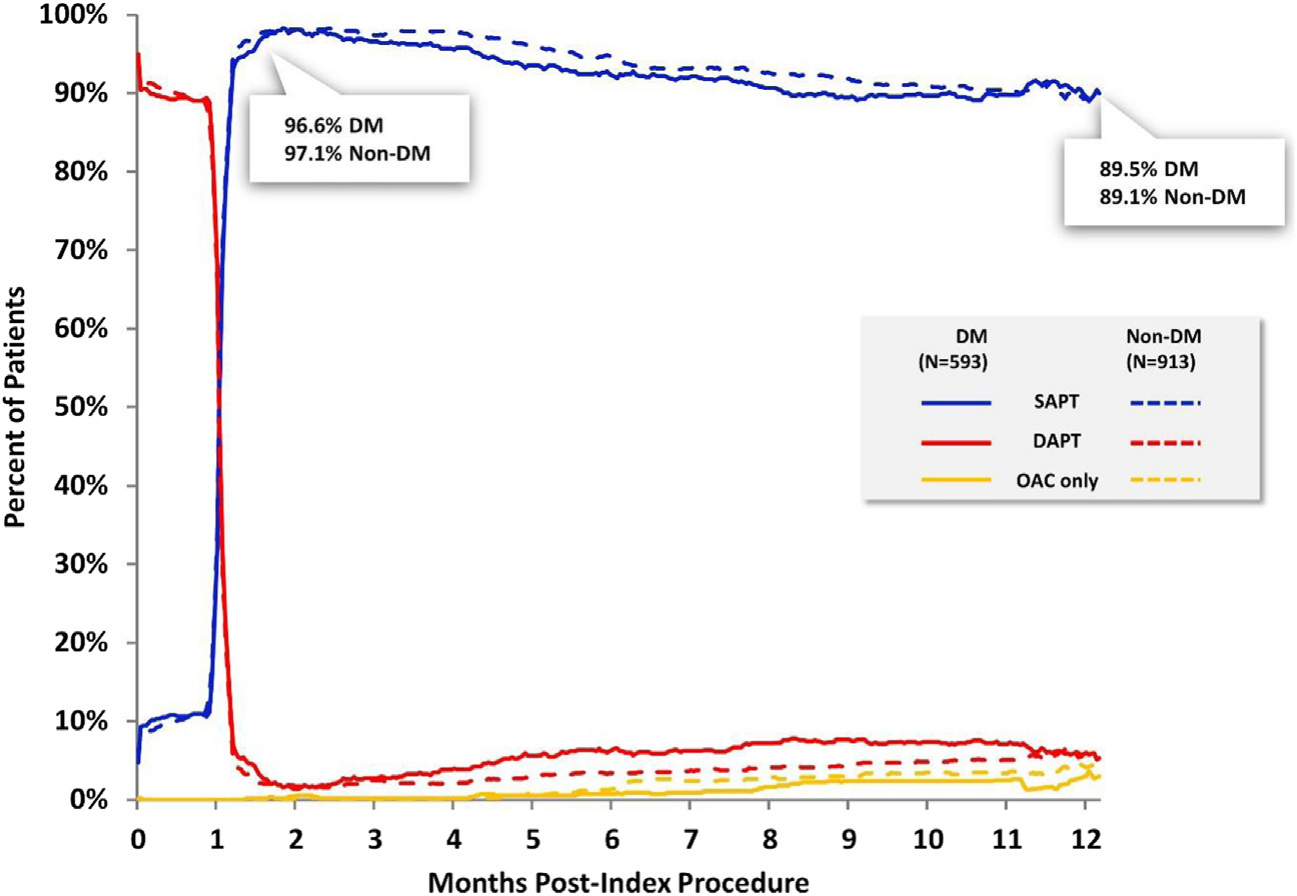
**DAPT, SAPT, and OAC usage in patients with DM versus without DM from procedure to 12 months.** DAPT, dual antiplatelet therapy; DM, diabetes mellitus; OAC, oral anticoagulant; SAPT, single antiplatelet therapy.

The mean number of HBR criteria was not significantly different between the 2 groups (Supplemental Table S1), although patients with DM were less likely to be aged ≥75 years compared with patients without DM, which was the most common HBR criteria met among the Onyx ONE Clear population. In contrast, patients with DM were nearly twice as likely to experience anemia (hemoglobin <11 g/dL) or receive transfusion (within 4 weeks before the procedure) and report renal failure (creatinine clearance <40 mL/min). Planned OAC continuation after PCI was similar between the groups. The discontinuation of DAPT at 1 month after PCI was similar between the groups, and 96.6% of patients with DM and 97.1% of patients without DM transitioned to SAPT by the second month (Figure 1). By 12 months, 89.5% of patients with DM were continued on SAPT, comparable with 89.1% of patients without DM.

### Outcomes

**Figure 2:**
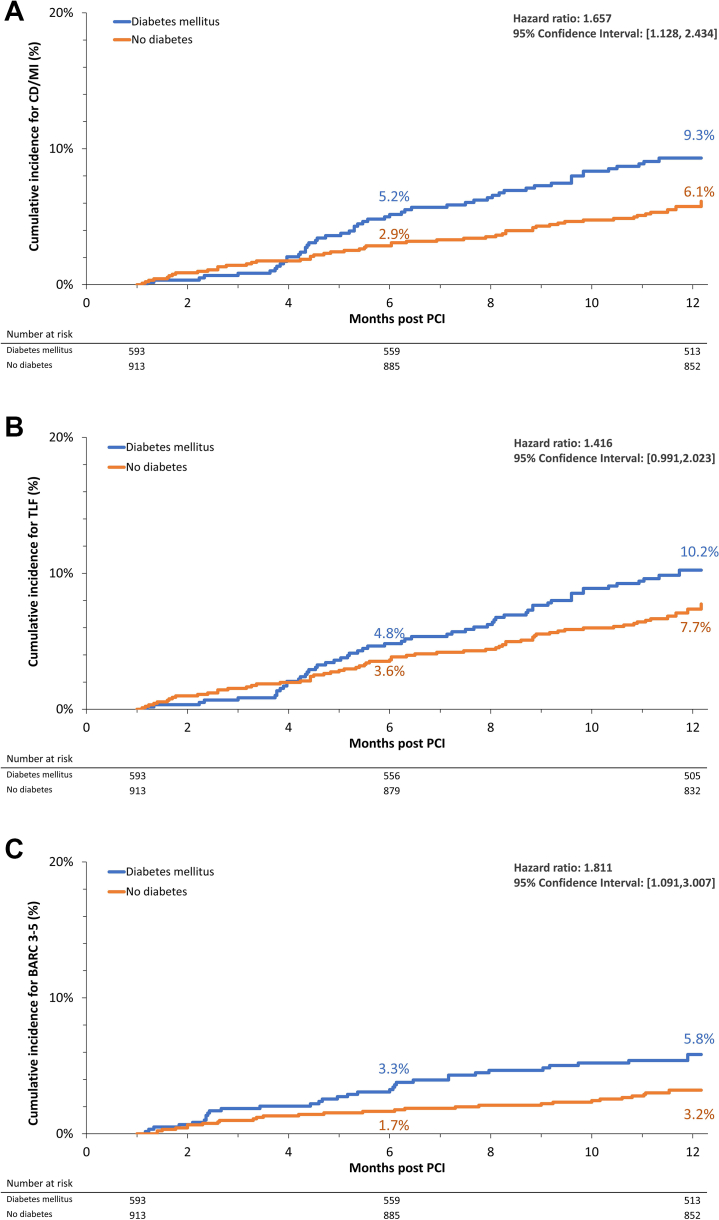
**Kaplan–Meier estimates of (A) CD/MI, (B) TLF, and (C) BARC 3-5 from 1 month through 12 months.** CD, cardiac death; MI, myocardial infarction; TLF, target lesion failure; BARC, Bleeding Academic Research Consortium.

The Kaplan-Meier rate estimates of the primary end point, the composite incidence of CD or MI between 1 month and 12 months, were significantly higher in patients with DM than those in patients without DM at 12 months (hazard ratio [HR], 1.657; 95% CI, 1.128-2.434) (Figure 2 and Supplemental Table S2). However, there were numerous clinical baseline differences between patients with diabetes and those without diabetes (Table 1). After propensity score adjustments for these differences, the composite rate of CD/MI was no longer significantly different between the groups (propensity score-adjusted HR, 1.374; 95% CI, 0.919-2.055; *P* = .122) (Table 2). The Kaplan-Meier rate of CD was numerically higher in patients with DM than that in patients without DM, but was not significantly different with or without propensity score adjustments (Table 3). The Kaplan-Meier rate of MI was significantly higher in patients with DM than that in patients without DM (*P* = .050); however, this was no longer significant after propensity score adjustment (*P* = .393).

**Table 3:** Kaplan–Meier rate estimates between 1 month and 12 months in patients with and without DM.

	DM (n = 593)	No DM (n = 913)	*P*, Cox regression	Propensity score-adjusted hazard ratio∗ (95% CI)	Propensity score-adjusted∗*P*, Cox regression
Primary end point: CD or MI	9.3 (53)	6.1 (51)	.010	1.374 (0.919-2.055)	.122
Death	8.9 (50)	4.5 (39)	<.001	1.909 (1.232-2.956)	.004
Cardiac death	3.8 (21)	2.1 (18)	.057	1.645 (0.852-3.179)	.138
Non-cardiac death	5.4 (29)	2.5 (21)	.007	2.135 (1.188-3.837)	.011
MI	6.3 (36)	4.4 (36)	.050	1.235 (0.761-2.003)	.393
Target vessel MI	5.3 (30)	4.3 (21)	.217	1.042 (0.625-1.736)	.874
Stent thrombosis (ARC definite/probable)	0.9 (5)	0.7 (5)	.475	1.073 (0.295-3.908)	.915
Target lesion failure	10.2 (57)	7.7 (64)	.056	1.222 (0.841-1.776)	.294
Target vessel failure	11.8 (64)	8.1 (67)	.016	1.330 (0.929-1.904)	.119
Clinically driven target lesion revascularization	4.3 (23)	3.4 (27)	.288	1.074 (0.601-1.920)	.809
Clinically driven target vessel revascularization	6.1 (31)	4.1 (33)	.110	1.268 (0.760-2.119)	.363
Stroke	1.2 (7)	1.7 (15)	.553	0.643 (0.254-1.631)	.353
Bleeding (BARC scale)					
BARC 1-5	15.6 (87)	12.5 (108)	.079	1.248 (0.930-1.675)	.140
BARC 2-5	13.8 (77)	11.4 (98)	.145	1.186 (0.869-1.619)	.282
BARC 3-5	5.8 (32)	3.2 (28)	.022	1.701 (1.001-2.891)	.050

Values are presented as % (n).

∗ The *P* values are obtained from a Cox regression analysis that was covariate adjusted using the propensity scores based on age, body mass index, previous percutaneous coronary intervention, previous coronary artery bypass grafting, hyperlipidemia, hypertension, atrial fibrillation, serum creatinine levels, multivessel coronary artery disease, worst Canadian Cardiovascular Society angina class, minimum baseline reference vessel diameter, and maximum lesion length as the confounding variables. ARC, Academic Research Consortium; BARC, Bleeding Academic Research Consortium; CD, cardiac death; DM, diabetes mellitus; MI, myocardial infarction.

Target lesion failure trended higher in the Kaplan-Meier rate estimate in patients with DM than that in patients without DM, but the difference was not significant (HR, 1.416; 95% CI, 0.991-2.023; *P* = .294) (Figure 2), even after propensity score adjustments (HR, 1.222; 95% CI, 0.841-1.776; *P* = .294) (Table 3). Conversely, target vessel failure and incidence of death were significantly higher in patients with DM than those in patients without DM (Table 3) but was no longer significantly different after propensity score adjustment for baseline differences. The difference in the incidence of death between the patient groups was partly because of a higher incidence of non-CD in patients with DM versus patients without DM (Table 3), although there remained a trend toward higher CD/MI in patients with DM. Notably, incidences of ST (definite/probable), clinically driven target lesion revascularization and target vessel revascularization, and stroke were not significantly different between patients with diabetes and those without diabetes as assessed by Kaplan-Meier rate estimates. Bleeding events (BARC 3-5) from 1 month through 12 months were more common in patients with versus those without DM (HR, 1.811; 95% CI, 1.091-3.007; *P* = .022) (Central Illustration), even after propensity score adjustment **(**HR, 1.701; 95% CI, 1.001-2.891; *P* = .050) (Table 3). However, renal failure (creatinine clearance <40 mL/min) and anemia (hemoglobin <11 g/dL) or blood transfusion within 4 weeks of PCI were bleeding risk criteria that were significantly higher in patients with DM than those in patients without DM (Supplemental Table S1) (18.4% vs 8.7%, *P* > .001 and 20.1% vs 10.7%, *P* < .001, respectively).

**Central Illustration:**
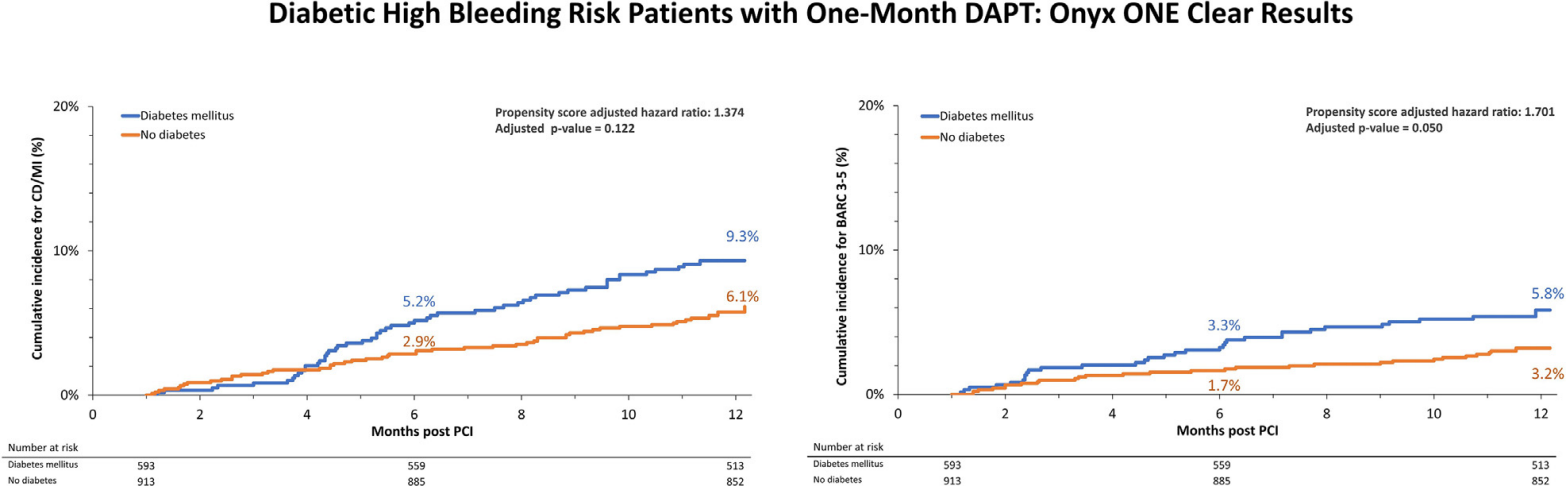
Diabetic, high bleeding risk patients with 1-month dual antiplatelet therapy (DAPT). DAPT after percutaneous coronary intervention (PCI) with Resolute Onyx was shown to be safe in patients with a high bleeding risk (HBR). Whether abbreviated DAPT is a suitable strategy also for HBR patients with high ischemic risk, such as diabetes mellitus (DM), is unknown. DM patients treated with 1-month DAPT after PCT had favorable 12-month ischemic outcomes (left), albeit with more bleeding events (right) compared with non-DM patients. These data support the safety and effectiveness of Resolute Onyx ZES followed by 1-month DAPT in HBR patients, including DM patients, considered to be at high ischemic risk.

The multivariable Cox regression analysis showed that renal failure and multivessel disease were predictors of the composite outcome of CD or MI from 1 month to 12 months after PCI (Table 4). Predictors of target lesion failure from 1 month to 12 months after PCI were renal failure, multivessel disease, and sex. Finally, predictors of BARC 3-5 bleeding were renal failure and anemia or blood transfusion with 4 weeks of PCI. Notably, DM was not a predictor of CD/MI, target lesion failure, and BARC 3-5 bleeding.

**Table 4:** Risk factors for the composite of cardiac death and myocardial infarction, target lesion failure, and BARC 3-5 in patients with DM versus without DM between 1 month and 12 months.

Parameter	Hazard ratio (95% CI)	*P*
Cardiac death or myocardial infarction		
Renal failure	1.93 (1.22-3.06)	.005
Multivessel disease	1.63 (1.09-2.43)	.018
Hypertension	1.95 (0.94-4.03)	.073
Diabetes mellitus	1.38 (0.93-2.05)	.108
Target lesion failure		
Renal failure	1.88 (1.21-2.94)	.005
Multivessel disease	1.57 (1.08-2.30)	.019
Female sex	0.65 (0.43-0.99)	.045
Smallest reference vessel diameter	0.69 (0.46-1.03)	.069
Diabetes mellitus	1.23 (0.85-1.77)	.272
BARC 3-5		
Renal failure	2.01 (1.08-3.75)	.028
Anemia or transfusion within 4 wk of PCI	1.88 (1.02-3.45)	.043
Hyperlipidemia	1.78 (0.92-3.44)	.085
Diabetes mellitus	1.51 (0.90-2.53)	.120

Stepwise selection was used to select significant risk factors from these variables: age (continuous), sex, hypertension, diabetes mellitus, body mass index, hyperlipidemia, previous myocardial infarction, previous percutaneous coronary intervention, previous coronary artery bypass grafting, previous stroke, a history of atrial fibrillation, acute coronary syndrome, Canadian Cardiovascular Society grading of angina pectoris II-IV, anemia or transfusion, renal insufficiency, multivessel disease, smallest reference vessel diameter, total lesion length (continuous variable), femoral vascular access, and complex percutaneous coronary intervention.^9^ A *P* value of <.1 was required to enter and a *P* value of >.1 was required to be removed, except diabetes mellitus, which was forced into the model.

BARC, Bleeding Academic Research Consortium.

## Discussion

Among the patients with HBR enrolled in the Onyx ONE Clear study, approximately 40% presented with DM. Despite patients with DM being younger, they were more likely to experience more comorbidities and receive longer stent lengths than those patients without DM. Clinical outcomes between 1 month and 12 months after treatment with the Resolute Onyx ZES during PCI followed by 1 month of DAPT were not favorable in patients with DM compared with those in patients without DM. However, after propensity score adjustments considering the numerous comorbidities associated with patients with DM in this study, treatment differences between the groups were mostly comparable (Table 3). These potentially favorable results after propensity score adjustments are notable given the fact that many patients enrolled in the Onyx ONE Clear study had a high ischemic risk and lesion complexity, including high rates of acute coronary syndrome (and ST-segment elevation MI), multivessel disease, renal failure, calcified lesions, and longer stents.^1^

Weighing the relative risks of ischemia and bleeding in patients with HBR after PCI with DES remains challenging. Recent European Society of Cardiology guidelines recommend DAPT for at least 6 months and 1 year after PCI in patients with stable coronary syndrome and acute coronary syndrome, respectively,^10^ with shortened DAPT for patients with HBR.^11^^,^^12^ Several meta-analyses comparing the relative risk of ischemic and bleeding events between short-term (≤6 months) and long-term (12 months) DAPT suggest that long-term DAPT increases the risk of bleeding out to 1 year; however, there is a disagreement on whether short-term DAPT necessarily increases the risk of ischemic-related events.^13^^,^^14^ Multiple studies, including the LEADERS FREE trial, demonstrated reasonable safety in reducing DAPT to 1 month after PCI in patients with HBR.^15^, ^16^, ^17^ In recently published results from the MASTER DAPT trial in which more than one-third of enrolled patients with HBR had DM, the 1-month DAPT was noninferior to the 3-month DAPT in the rate of ischemic events over 335 days and reduced major bleeding.^18^

Evaluating ischemic versus bleeding risks in patients with diabetes at HBR is an even greater challenge because DM is associated with an increase in ischemic^19^, ^20^, ^21^ and bleeding events.^22^^,^^23^ Meta-analyses of patients with diabetes after PCI treated with DES comparing short-term (≤6 months) and long-term (12 months) DAPT found that long-term DAPT increased the risk of bleeding without providing a benefit to patients with DM in reducing the risk of ischemic events.^24^^,^^25^ However, only a small proportion of patients in these trials experienced acute coronary syndrome and/or complex PCI. Before the analysis reported in this study, it was unclear whether DAPT as short as 1-month after DES implantation was a suitable treatment option to safely manage ischemic versus bleeding risk in patients with diabetes at HBR.

One month after PCI, patients with and without DM discontinued DAPT and transitioned to SAPT at similar rates, and although the rate of BARC 3-5 bleeding events was higher in patients with diabetes, the reassuring ischemic outcomes observed between patients with and without DM after propensity score adjustments suggested that ZES implantation followed by 1-month DAPT is safe and effective for patients with DM at HBR. The increased bleeding risk in patients with diabetes is tempered by the fact that renal failure and anemia are independent risk factors of bleeding, both of which are much more common in patients with DM than in patients without DM. After adjusting for baseline and clinical procedural differences between the groups, safety and efficacy outcomes, including CD, target vessel–related MI, ST, and target lesion revascularization, were not significantly different for patients with and without DM at HBR. Death rates remained significantly higher in patients with DM, however, primarily driven by increased non-CDs, indicative of the morbidity risks associated with DM. Collectively, these data support the safety and efficacy of the Resolute Onyx ZES followed by 1-month DAPT in patients with DM at HBR.

### Limitations

First, the Onyx ONE Clear study was a single-arm study in which all patients received 1-month DAPT; thus, shorter or longer DAPT durations were not evaluated in this analysis. This prespecified analysis was not powered to detect low frequency events, such as ST, between patients with and without DM. Second, outcomes were assessed only in patients who were event free during 1 month after PCI. Finally, because only patients receiving the Resolute Onyx ZES were included in the Onyx ONE Clear study, this analysis cannot necessarily be extended to other DESs.

## Conclusions

A large proportion of patients at HBR enrolled in the Onyx ONE Clear study presented with DM. Despite differences in procedural and baseline patient characteristics, patients with DM treated with 1-month DAPT after PCI had favorable 12-month ischemic outcomes, albeit with more bleeding events than those in patients without DM. These data support the safety and effectiveness of the Resolute Onyx ZES followed by 1-month DAPT in patients with HBR, including patients with DM, considered to be at a high ischemic risk.
