# Supplementary material for: Patients With Diabetes at High Bleeding Risk With 1-Month Dual Antiplatelet Therapy: Onyx ONE Clear Results
Source: J Soc Cardiovasc Angiogr Interv. 2022 Aug 25;1(5):100441. doi: 10.1016/j.jscai.2022.100441 (PMC11307822; doi:10.1016/j.jscai.2022.100441)
Supplement: Supplementary Material 1 [file mmc1.docx]

**Supplement for Kedhi *et al.* Diabetic High Bleeding Risk Patients with One-Month DAPT**

**Supplemental Table S1: Baseline HBR criteria**

| **mean ± SD or % (N)** | **DM (N=593)** | **No DM (N=913)** | **p-value** |
| --- | --- | --- | --- |
| Mean HBR criteria | 1.6 ± 0.8 | 1.5 ± 0.7 | 0.059 |
| Oral anticoagulation to continue after PCI | 38.4 (228) | 42.6 (389) | 0.120 |
| Age ≥75 years | 53.5 (317) | 62.7 (572) | < 0.001 |
| Hgb <11 g/dl (or transfusion within 4 weeks before procedure) | 20.1 (119) | 10.7 (98) | < 0.001 |
| Creatinine clearance <40 ml/min | 18.4 (109) | 8.7 (79) | < 0.001 |
| Non skin cancer diagnosed or treated within 3 years | 6.4 (38) | 8.1 (74) | 0.229 |
| Planned surgery in next 12 months requiring interruption of DAPT | 6.2 (37) | 6.9 (63) | 0.672 |
| Expected non-compliance to prolonged DAPT | 5.1 (30) | 3.7 (34) | 0.239 |
| Hospital admission for major bleeding in prior 12 months | 3.9 (23) | 2.1 (19) | 0.053 |
| NSAID (other than aspirin) or steroids for ≥ 30 days after PCI | 3.4 (20) | 3.0 (27) | 0.652 |
| Stroke in previous 12 months | 2.5 (15) | 2.6 (24) | 1.000 |
| Thrombocytopenia (PLT <100,000/mm^3^) | 2.2 (13) | 1.4 (13) | 0.312 |
| Prior intracerebral bleed | 1.9 (11) | 1.6 (15) | 0.840 |
| Severe chronic liver disease | 1.0 (6) | 0.9 (8) | 0.790 |

HBR: high bleeding risk, PCI: percutaneous coronary intervention, Hgb: hemoglobin, DAPT: dual antiplatelet therapy, PLT: platelets

**Supplemental Table S2: Clinical outcomes between 1 to 12 months in patients with and without DM**

|  | **DM**  **(N=536)** | **No DM**  **(N=970)** | **Unadjusted p-value** | **Propensity score-adjusted difference* [95% CI]** | **Adjusted p-value** |
| --- | --- | --- | --- | --- | --- |
| Primary endpoint: Cardiac death or MI | 9.1% | 5.6% | 0.012 | 2.4% [-0.4%, 5.2%] | 0.094 |
| Death | 8.6% | 4.3% | 0.001 | 4.0% [1.4%, 6.7%] | 0.002 |
| - Cardiac death | 3.6% | 2.0% | 0.067 | 1.3% [-0.3%, 3.0%] | 0.116 |
| - Non-cardiac death | 5.0% | 2.3% | 0.007 | 2.7% [0.6%, 4.8%] | 0.012 |
| Myocardial infarction | 6.2% | 4.0% | 0.063 | 1.2% [-1.1%, 3.6%] | 0.302 |
| Target vessel myocardial infarction | 5.1% | 3.9% | 0.244 | 0.3% [-1.8%, 2.5%] | 0.762 |
| Stent thrombosis (ARC), definite/probable | 0.9% | 0.6% | 0.525 | 0.0% [-0.8%, 0.9%] | 0.936 |
| Target lesion failure (TLF) | 9.8% | 7.0% | 0.065 | 1.7% [-1.2%, 4.7%] | 0.252 |
| Target vessel failure (TVF) | 11.0% | 7.4% | 0.019 | 2.5% [-0.6%, 5.7%] | 0.110 |
| Clinically driven target lesion revascularization (TLR) | 3.9% | 3.0% | 0.307 | 0.2% [-1.8%, 2.2%] | 0.808 |
| Clinically driven target vessel revascularization (TVR) | 5.3% | 3.6% | 0.149 | 1.0% [-1.3%, 3.3%] | 0.408 |
| Stroke | 1.2% | 1.7% | 0.519 | -0.6% [-2.0%, 0.8%] | 0.400 |
| MACE^†^ | 15.1% | 9.5% | 0.001 | 4.5% [0.9%, 8.1%] | 0.014 |
| Bleeding, BARC scale |  |  |  |  |  |
| BARC 1-5 | 14.9% | 11.9% | 0.098 | 3.1% [-0.6%, 6.7%] | 0.102 |
| BARC 2-5 | 13.2% | 10.8% | 0.162 | 2.2% [-1.3%, 5.7%] | 0.210 |
| BARC 3-5 | 5.5% | 3.1% | 0.030 | 2.3% [0.0%, 4.5%] | 0.050 |

^*^The p-values are adjusted by stratification/subclassification using 5 groups determined by quintiles propensity scores, based on age, BMI, Previous PCI, Previous CABG, Hyperlipidemia, Hypertension, Atrial Fibrillation, Serum Creatinine, Multivessel Coronary Artery Disease, Worst Canadian Cardiovascular Society Angina Class, Minimum Baseline RVD and Maximum Lesion Length
